# *Nocardia farcinica* as a cause of chronic meningitis – case report

**DOI:** 10.1186/s12879-020-4764-y

**Published:** 2020-01-17

**Authors:** Anna Moniuszko-Malinowska, Piotr Czupryna, Izabela Swiecicka, Henryk Grześ, Agnieszka Siemieniako, Sambor Grygorczuk, Eugeniusz Tarasów, Sławomir Pancewicz

**Affiliations:** 1 Department of Infectious Diseases and Neuroinfections, Medical University of Białystok, Zurawia 14; 15-540, Bialystok, Poland; 2 Department of Microbiology, University of Bialystok, Ciołkowskiego 1J, 15-245, Bialystok, Poland; 3 Microbiology Laboratory, University Hospital in Białystok, Zurawia 14; 15-540, Bialystok, Poland; 4 Department of Radiology, Medical University of Białystok, Białystok, Poland

**Keywords:** *Nocardia farcinica*, Meningitis

## Abstract

**Background:**

Nocardiosis is an uncommon disease caused by aerobic gram-positive bacteria *Nocardia* spp. Although it is usually an opportunistic infection affecting immunocompromised patients, even one third of cases occur in immunocompetent persons. The aim of the study was to describe the course of chronic meningitis due to *Nocardia* infection.

**Case presentation:**

A 52-year-old patient, chalk miner, suffered from a chronic meningitis caused by an extremely rare pathogen. The patient’s history was complicated and diagnostic process covered multiple examinations and consultations. Initially *Kocuria rosea* was cultured, yet after molecular examination the result was verified to *Nocardia farcinica.* Targeted antibiotic treatment was implemented, which resulted in gradual improvement of patients condition. A full recovery was achieved after one year antibiotic therapy.

**Conclusions:**

1. *Nocardia farcinica* is an uncommon but possible cause of chronic meningitis.
2. In the case of a chronic meningitis of unknown origin multiple cerebrospinal fluid cultures should be performed as the identification of pathogen may be crucial for patient’s recovery.
3. In case of unusual culture, such as *Kocuria* spp. PCR should be performed.

## Background

Nocardiosis is an uncommon disease caused by aerobic gram-positive bacteria *Nocardia* spp. [1]. It is an opportunistic infection, which usually affects immunocompromised patients, however one third of patients with nocardiosis may be immunocompetent. Nocardiosis may present as a single organ or multifocal disease. The single organ infection most commonly manifests as a lung disease (39% of hospitalized patients) or CNS disease (9% of patients). The aim of the study was to describe the course of chronic *Nocardia* meningitis.

### Case report

A 52-year-old patient, chalk miner, was admitted to the regional hospital for investigation of sa 2-week history of severe headache and fever. Physical examination revealed fever, pain on palpation in cervical and lumbosacral region, and meningeal signs. Features suggestive of bacterial meningitis were detected in the cerebrospinal fluid. The empirical antibiotic therapy with ceftriaxone and vancomycin was implemented. Subsequently vancomycin was replaced with ampicillin. CSF and blood cultures were negative (Table 1).

**Table 1:** Results of CSF examinations

Data	Pleocytosis (cells/μl)	Lymphocytes (%)	Granulocytes (%)	Monocytes (%)	Protein concentration (mg/dL)	Glucose concentration (mg/dL)	CSF culture
02.02.2015	2240	–	–	–	1609	13	No growth
09.02.2015	246	53	43	4	125.7	26	No growth
20.02.2015	135	90	3	7	83.4	39	No growth
03.03.2015	2770	9	88	3	159	24	Growth of *Kocuria* spp.,
16.03.2015	1463	20	80	–	146.1	34	Growth of *Kocuria* spp.,
27.03. 2015	1731	10	85	5	169.9	16	Growth of *Kocuria* spp., finally diagnosed as *Nocardia farcinica* with PCR
07.04.2015	1438	16	84	–	159.6	26	No growth
20.04.2015	460	10	90	–	194.6	21	No growth
20.06.2015	350	–	–	–	166.4	–	No growth
18.08.2015	191	22	73	5	131.9	31	No growth
07.10.2015	15	–	–	–	46.6	53	No growth
05.01.2016	12	58	42	–	32.7	53	No growth

**Fig. 1:**
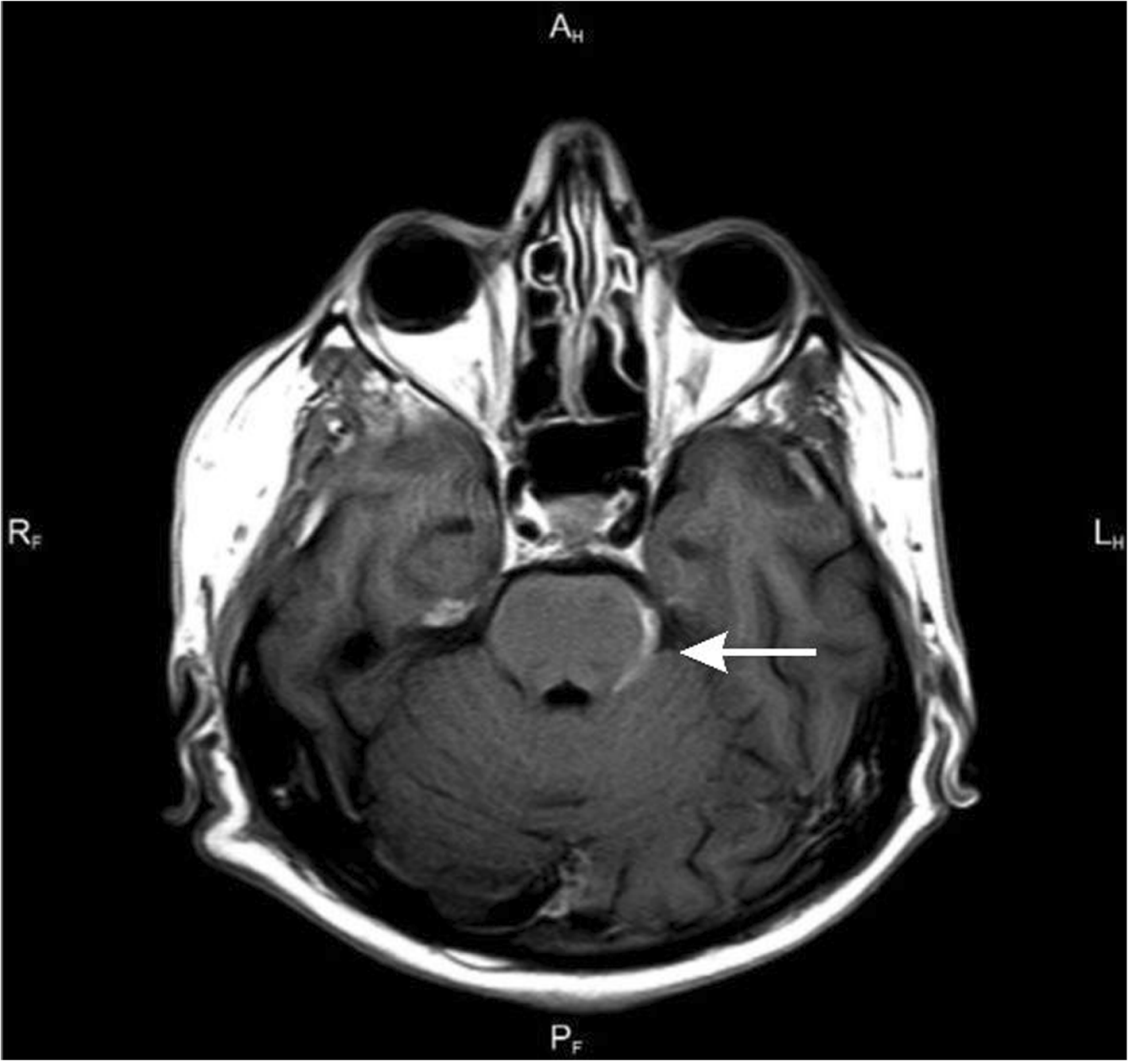
Axial T1-weighted scan after contrast enhancement - contrast enhancement of the left lateral surface of the pons

**Fig. 2:**
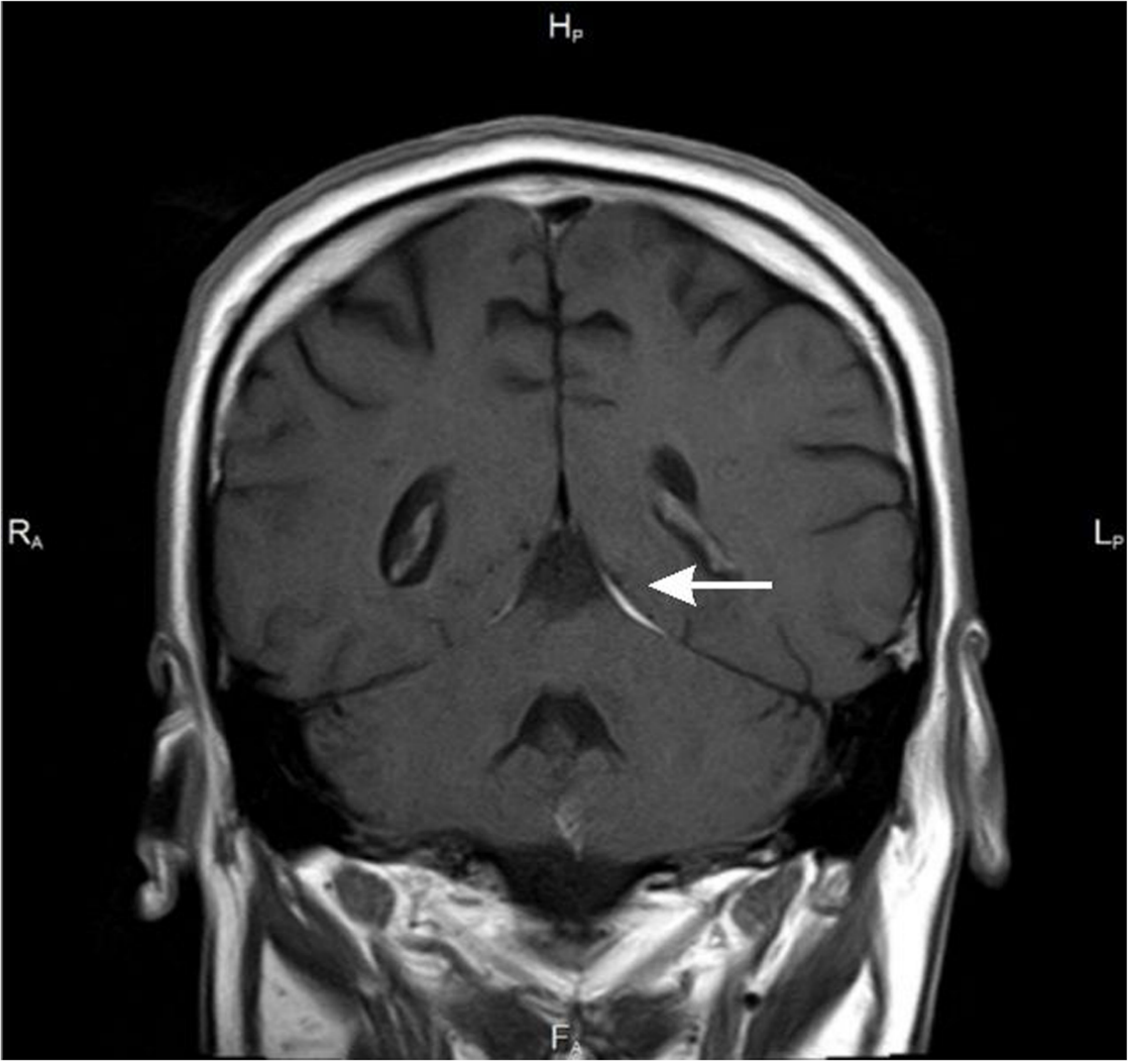
Coronal T1-weighted scan after contrast enhancement - contrast enhancement is also visible in the cerebellar tentorium on the left

**Fig. 3:**
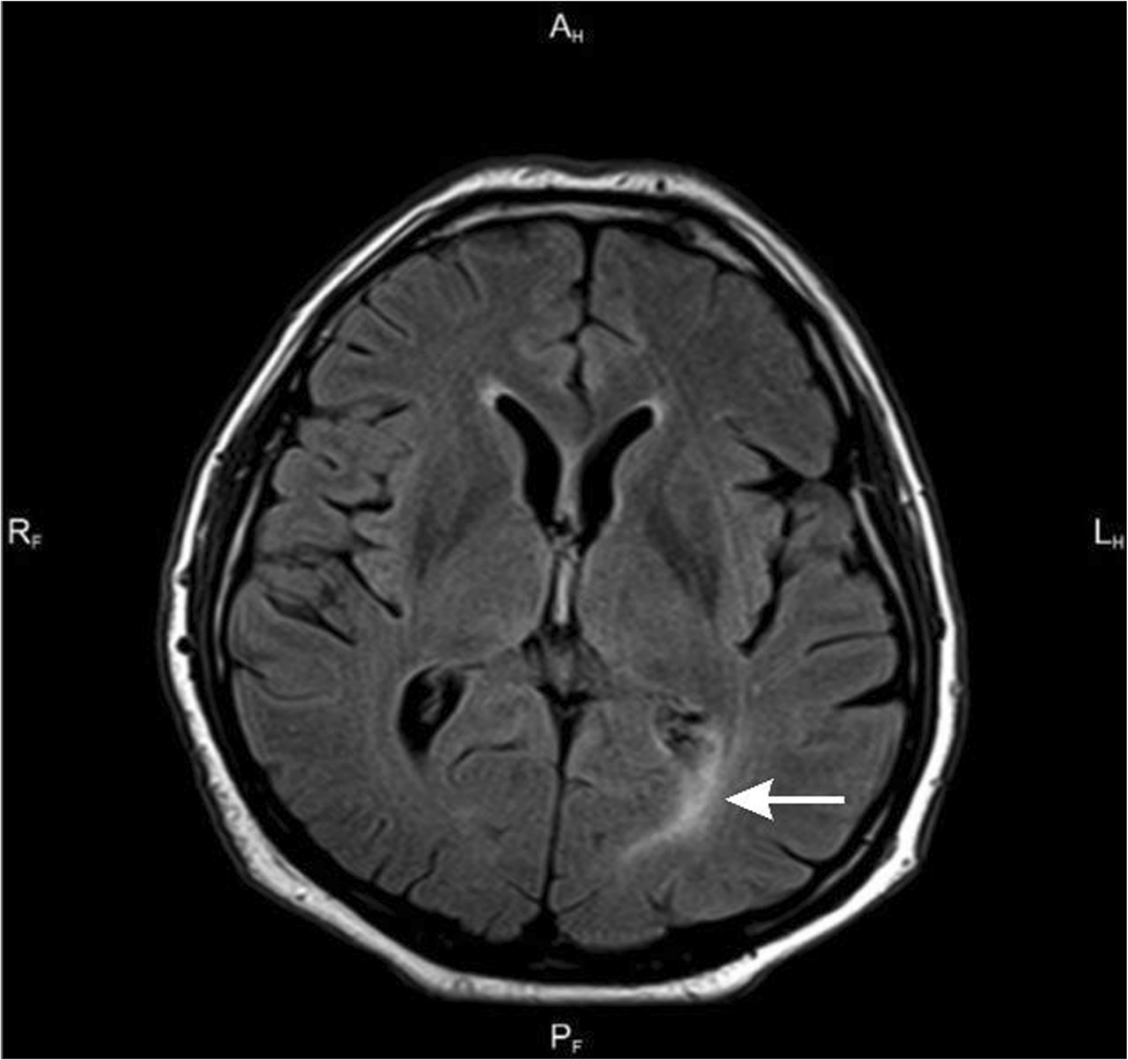
Axial FLAIR scan

**Fig. 4:**
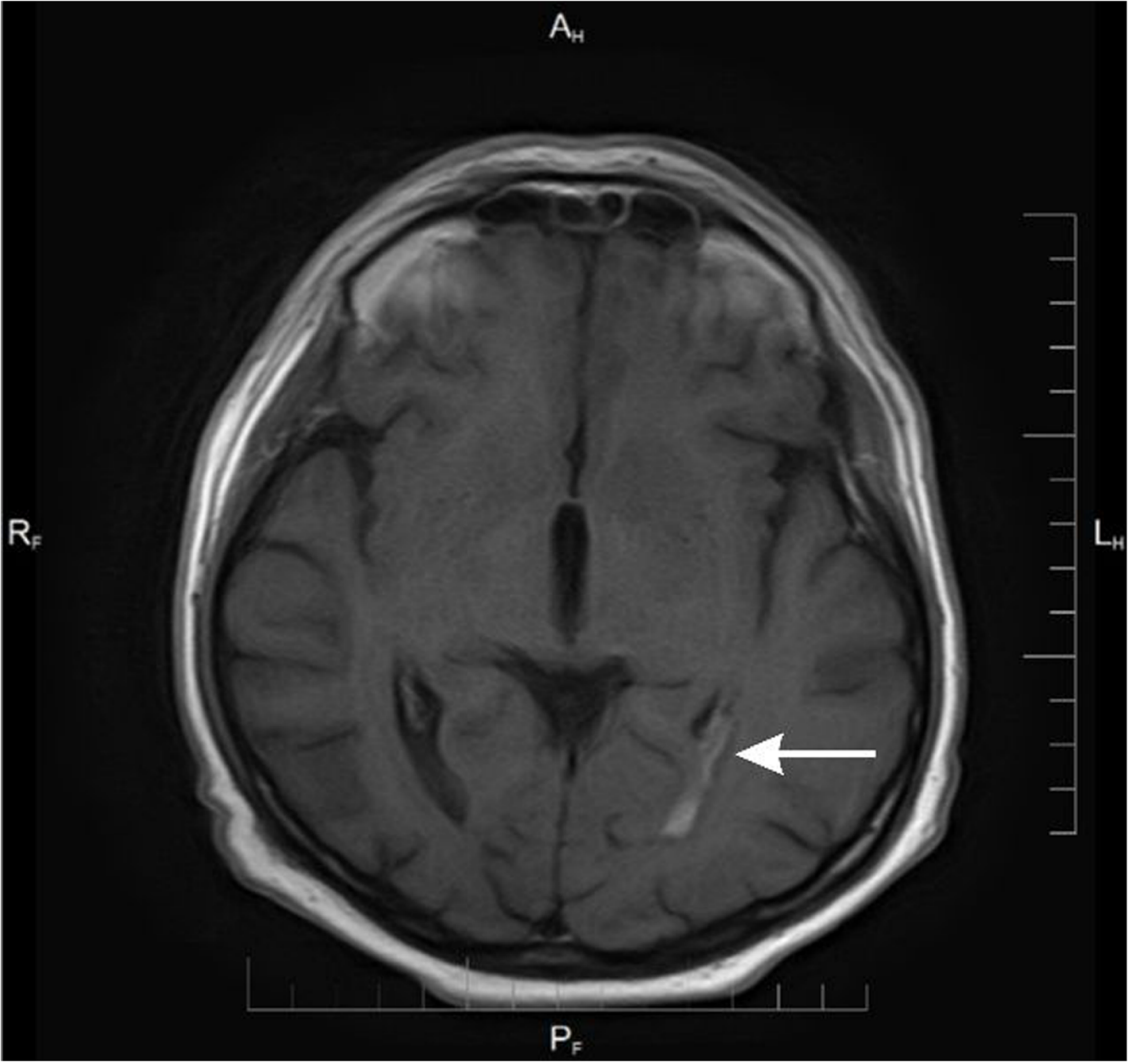
Axial T1-weighted scan after contrast enhancement - Enhancing lesion in choroid plexus of the left occipital temporal horn of the lateral ventricle with surrounding edema visible in FLAIR scan

Because of persisting headache in spite of the treatment, MRI of the brain was performed (Table 2; Figs. 1, 2, 3 and 4).

**Table 2:** Results of imaging tests

Data	Brain MRI results
30.12. 2014	- Widening of the posterior anchor of the left lateral - Ventriculitis - Widened Robin-Virchoff Virchow-Robin area on the left side
13.03.2015	- Inflammatory enhancement of meninges - Inflamatory lesions in the anterior part of cereberral tent - Asymmetry of occipital horns (Figs. 1, 2, 3 and 4)
06.10.2015	- Regression of lesions - Lesions in occipital horns similar to the previous examination
16.03.2016	- Further regression of lesions - Post inflammatory changes

Patient was consulted by a dentist – decayed teeth and maxillary cysts were observed. The cyst was surgically removed and sanitation of oral cavity was performed. *Enterobacter cloacae* was cultured from the cyst, followed with meropenem and amikacin treatment according to the antibiogram. Because of the persisting symptoms the patient was transferred to the Department of Infectious Diseases and Neuroinfections, Medical University of Bialystok, Poland.

Antibiotic therapy was continued. Patient received meropenem, aminoglycosides, ciprofloxacin, which resulted with remission of fever and headache, as well as meningeal signs and pain on palpation in cervical and lumbosacral region. However, with the antibiotic withdrawal the symptoms reappeared. Additionally, cerebellar symptoms and sensory disturbances appeared.

Multidirectional diagnostic protocol was implemented: latex test of CSF (*Neisseria meningitidis* B, *E.coli* K1 (−); *Haemophilus influenzae* b(−); *Streptococcus pneumoniae* (−); *Streptococcus* B (−); *Neisseria meningitidis* A (−); *Neisseria meningitidis* C (−); *Neisseria meningitidis* Y/W 135 (−) Pastorex Meningitis; BIORAD), syphilis tests, bacterial and fungi cultures, DNA hybridization test and culture for *Mycobacterium tuberculosis*, multiplex-PCR CSF for viruses (HSV-1, HSV-2, VZV, EBV, CMV, HHV-6), CSF cytology. All results were negative. The patient was consulted by otolaryngologist, ophthalmologist, neurosurgeon, dentist, and psychiatrist. Numerous neuroimaging examinations (US, X-ray, CT, MRI) were performed (Table 2).

Subsequent CSF cultures were negative (Table 1). Finally in the 4th, 5th and 6th CSF culture, Gram-positive cocci were cultured. Whitish, small, round, raised, convex colonies of 2–3 mm size on initial isolation and non-diffusible yellowish pigmentation after prolonged incubation were observed. Initially with the use of GP identification cards and automatic systems VITEK 2 (bioMerieux) the bacteria were identified as *Kocuria* spp.

In the molecular examination, genomic DNA of the bacteria was extracted from an overnight culture in a brain-heart infusion (BHI) broth using the DNeasy Blood & Tissue Kit (Qiagen GmbH, Hilden, Germany) with the protocol for Gram-positive bacteria. The DNA concentration and its purity were assessed in the NanoDrop 2000 spectrometer (Thermo Fisher Scientific Com., Waltham, USA). Then, a pair of primers 27F 5′-AGAGTTTGATCMTGGCTCAG-3′ and 1525R 5’AAGGAGGTGWTCCRCC-3′ [2] were used to amplified the 16S rRNA gene of the isolate in PCR performed in the Veriti 96 Well thermal cycler (Applied Biosystems, Foster City, USA) at the following conditions:: 94 °C for 3 min, 30 cycles 94 °C for 30 s, 56 °C for 45 s, and 72 °C for 90 s, and the final elongation for 7 min at 72 °C. The PCR product was analyzed in a gel electrophoresis and purified with the QiaAmp PCR purification kit (Qiagen). The sequencing reaction was performed using the Big Dye Terminator cycle sequencing kit (Applied Biosystems); the product was purified using the ExTerminator Kit (A&A Biotechnology) and sequenced with the ABI3500 automated sequencer (Applied Biosystems). The sequences were assembled with the BioEdit Sequence Alignment Editor version 7.0.1. For the comparative analyses of nucleotide and amino acid sequences, database searches were performed using the BLAST programs at the NCBI website (http://www.ncbi.nlm.nih.gov). This study allowed verification of the initial identification to *Nocardia farcinica*.

Treatment with trimethoprim / sulfamethoxazole was initiated, resulting in slow improvement of patient’s condition, regression of cerebellar symptoms and sensory disturbances, and decrease in the inflammatory parameters of the CSF. The total duration of treatment was 1 year and resulted in complete recovery.

## Discussion and conclusions

*Nocardia* spp. is present in soil and may be transmitted through direct inhalation of contaminated particles. In case of our patient, working in a chalk mine might therefore be considered a risk factor of *Nocardia* spp. infection.

Nocardiosis usually presents as a self-limiting respiratory tract infection. However, in some patients *Nocardia* spreads from lungs to other organs with a particular affinity for the brain. The most common neurological symptoms in the course of nocardiosis are: focal neurologic abnormalities, altered mental status, seizures, visual changes, ataxia. Nocardial meningitis is an infrequent manifestation of CNS nocardiosis and can occur with or without an associated brain abscess [3]. In our patient the disease presented initially only with fever and headache, with cerebellar syndrome and sensory disturbances appearing later in the course of the infection.

As *Nocardia* spp. are relatively slow-growing bacteria that can be challenging to recover, multiple CSF specimens should be cultured to increase the yield, although it is not uncommon for the bacteria to be recovered only when direct pus is cultured [4]. In routine aerobic cultures, *Nocardia* spp. have variable colonial morphology, from chalky white to pigment-producing orange, yellow, or brown colonies [5]. The preferred methods for speciation of *Nocardia* are 16S rRNA gene analysis and other molecular techniques, such as restriction fragment length polymorphisms and multilocus sequence analysis.

In our case the 4th culture was at first mistaken for *Kocuria rosea*, which morphologically resembles *Nocardia*. Only the following molecular diagnosis allowed us to properly identify the pathogen and adjust the treatment. Only molecular diagnosis differentiated *Nocardia farcininca* from *Kocuria rosea.*

Most authorities recommend trimethoprim-sulfamethoxazole (TMP-SMX) as part of first-line therapy for nocardiosis [6]. In case of our patient treatment with TMP-SMX resulted with a complete recovery.

Based on our experience we concluded, that:

1. *Nocardia farcinica* is an uncommon but possible cause of chronic meningitis.
2. In the case of chronic meningitis of unknown origin multiple cerebrospinal fluid cultures should be encouraged as the identification of pathogen may be crucial for patient’s recovery.
3. In case of unusual culture, such as *Kocuria* spp. PCR should be performed.

## Abbreviations

CMV: Cytomegalovirus
CSF: Cerebrospinal fluid
CSN: Central nervous system
CT: Computer tomography
EBV: Epstein barr virus
HHV-6: Human herpes virus – 6
HSV-1, HSV-2: Herpes simples virus − 1; 2
MRI: Magnetic resonance
TMP-SMX: Trimethoprim-sulfamethoxazole
US: Ultrasonography
VZV: Varicella zoster virus
